# Oridonin Suppresses Bladder Cancer Growth and Metastasis by Inducing S-Phase Arrest and Apoptosis

**DOI:** 10.3390/ijms27156835

**Published:** 2026-07-30

**Authors:** Wenqiang Sun, Yongchao Li, Menglong Xu, Haocheng Guan, Tinghui Wu, Shuwei Li

**Affiliations:** 1 The State Key Laboratory Incubation Base for Conservation and Utilization of Bio-Resource in the Tarim Basin of the Xinjiang Production and Construction Corps, Department of Biochemistry and Molecular Biology, College of Life Science and Technology, Tarim University, Alar 843300, China; swq9577@163.com (W.S.); 18850569602@163.com (Y.L.); xumenglong1989@163.com (M.X.); ghc13703476155@163.com (H.G.); 2 School of Medical Technology, Xinjiang Hetian College, Hetian 848000, China

**Keywords:** bladder cancer, oridonin, transcriptomics, proteomics, apoptosis, cell cycle

## Abstract

Bladder cancer (BC) remains a major clinical challenge owing to limited therapeutic options and high recurrence rates. Oridonin (ORI), a natural diterpenoid derived from *Rabdosia* plants, exhibits promising anti-tumor activity, but its effects and mechanisms in BC remain poorly defined. We evaluated the anti-BC potential of ORI in vitro and in vivo using proliferation, migration, invasion, cell-cycle, and apoptosis assays, integrated transcriptomic and proteomic analyses, Western blotting, and a 5637 xenograft model. ORI dose- and time-dependently inhibited 5637 and T24 cell proliferation, induced S-phase arrest (from 23.37% to 42.12% in 5637 and from 31.87% to 50.28% in T24; *p* < 0.01), and reduced migration (from 53.39% to 13.77% and from 59.81% to 12.49%; *p* < 0.0001) and invasion (from 74.42% to 25.43% and from 67.03% to 30.12%; *p* < 0.001). Multi-omics analyses revealed widespread changes enriched in apoptosis- and cell-cycle-related pathways. Consistently, ORI promoted apoptosis and necrosis, up-regulating BAX, CASP3, BID, and CYCS and down-regulating BCL2, validating the omics findings. In vivo, ORI (20 mg/kg, daily gavage) significantly suppressed xenograft growth (*p* < 0.001) without obvious toxicity, indicating that ORI inhibits BC growth and metastasis by inducing S-phase arrest and apoptosis and is a promising candidate for BC therapy.

## 1. Introduction

Bladder cancer (BC) is a significant global health challenge and ranks as the ninth most commonly diagnosed malignancy worldwide. According to the 2022 estimates of the International Agency for Research on Cancer (IARC), BC accounted for approximately 614,000 new cases and 220,000 deaths globally [1,2]. The nonspecific nature of early clinical manifestations contributes substantially to diagnostic and therapeutic delays, so the disease often remains undetected until local invasion or metastasis has occurred [3,4]. Once the tumor invades the muscular layer of the bladder or metastasizes to distant sites such as the lungs and bones, patient prognosis deteriorates markedly, with limited therapeutic options available [5,6]. Although systemic chemotherapy remains a cornerstone in the management of advanced BC, its efficacy is frequently constrained by drug toxicity and the development of resistance [7,8]. Therefore, identifying new therapeutic agents with improved safety and efficacy profiles is of critical importance.

Oridonin (ORI) is a diterpenoid compound derived from the traditional herbal medicine *Rabdosia rubescens* [9,10,11,12]. It exhibits a broad spectrum of pharmacological activities, including anti-tumor, anti-inflammatory, anti-microbial, and neuromodulatory effects [13,14,15,16,17,18]. ORI shows inhibitory activity against a variety of tumor cells, including gastric cancer cells, human laryngeal cancer cells, lung cancer cells, and rectal cancer cells [19,20]. Notably, ORI can inhibit the proliferation of gastric cancer SGC-7901 cells by regulating the TNF-α/AR/TGF-β signaling axis and can significantly reduce the tumorigenicity of Hep-2 cells in nude-mouse xenograft models [21,22,23]. Although ORI has shown good inhibitory effects on a variety of tumor cells, its role in bladder cancer has only recently begun to be explored [24], and its effects on cell-cycle regulation and apoptosis in BC remain poorly defined. Therefore, this study aims to investigate the effect of ORI on bladder cancer cells, with a particular focus on its influence on cell apoptosis and cell-cycle regulation.

## 2. Results

### 2.1. Anti-Proliferative Activity of ORI Against Human Bladder Cancer Cells 5637 and T24 In Vitro

The capacity of ORI to inhibit the proliferation of 5637 and T24 cells was evaluated using CCK-8 assays to establish IC50 values. When the cells were treated with different concentrations of ORI (0, 2.5, 5, 10, 12.5, 15, 17.5 and 20 μM) for 24 and 48 h, cell viability decreased in a time- and concentration-dependent manner. Specifically, the IC50 values after 24 h of treatment were 8.142 μM for 5637 cells and 9.359 μM for T24 cells. After 48 h of treatment, the IC50 values decreased to 6.128 μM for 5637 cells and 6.225 μM for T24 cells (Figure 1B,C).

### 2.2. ORI Induces S-Phase Cell-Cycle Arrest in 5637 and T24 Bladder Cancer Cells

To evaluate the effect of ORI on the cell cycle, 5637 and T24 cells treated with ORI at the 24-h IC50 (8.142 μM for 5637 and 9.359 μM for T24) for 24 h were stained with propidium iodide (PI) and analyzed by flow cytometry (Figure 2A,C). In 5637 cells, the proportion of cells in S phase increased significantly from 23.37% to 42.12% (*p* < 0.01), whereas the G1 population decreased from 67.23% to 35.84% and the G2 population increased slightly from 9.40% to 22.03% (Figure 2B). Similarly, in T24 cells the S-phase proportion increased from 31.87% to 50.28% (*p* < 0.01), accompanied by a decrease in the G1 population from 58.47% to 35.20% and a minor increase in the G2 population from 9.65% to 14.52% (Figure 2D).

### 2.3. ORI Inhibits Bladder Cancer Cell Migration and Invasion

To evaluate the effect of ORI on the migratory capacity of bladder cancer cells, a wound-healing assay was performed. After 24 h of ORI treatment at the 24-h IC50 (8.142 μM for 5637; 9.359 μM for T24), the migration rate of 5637 cells decreased from 53.39% in the control group to 13.77% in the ORI-treated group (*p* < 0.0001, a 74.2% reduction; Figure 3A,B). Likewise, the migration rate of T24 cells decreased from 59.81% to 12.49% (*p* < 0.0001, a 79.1% reduction; Figure 3C,D). The migration (wound-closure) rate was calculated as (A0 − A24)/A0 × 100%, where A0 and A24 denote the scratch areas at 0 and 24 h, respectively.

To further explore the anti-metastatic potential of ORI, Transwell assays were performed to analyze its effect on the invasive capacity of bladder cancer cells. ORI was applied at the 24-h IC50 (8.142 μM for 5637; 9.359 μM for T24) for 24 h. In 5637 cells, the invasion rate decreased from 74.42% in the control group to 25.43% in the ORI-treated group (*p* < 0.001, a 65.8% reduction; Figure 4A,B). In T24 cells, control cells exhibited strong invasive ability, whereas the invasion rate decreased from 67.03% to 30.12% after ORI treatment (*p* < 0.001, a 55.1% reduction; Figure 4C,D). The invasion rate was calculated as the percentage of invaded cells relative to the control group.

### 2.4. ORI Promotes Apoptosis and Necrosis in Bladder Cancer Cells In Vitro

Apoptosis and necrosis in 5637 and T24 cells after 24 h of ORI treatment at the 24-h IC50 (8.142 μM for 5637; 9.359 μM for T24) were assessed by YO-PRO-1/PI staining. Relative to controls, ORI-treated 5637 and T24 cells exhibited a substantially greater proportion of YO-PRO-1-positive cells (indicating early apoptosis; green fluorescence) and PI-positive cells (indicating late apoptosis or necrosis; red fluorescence). In particular, ORI exposure visibly intensified YO-PRO-1 staining in both cell lines, suggesting the induction of early-stage apoptosis (Figure 5). The increased PI staining in ORI-exposed cells further implied a rise in late apoptosis and/or necrotic cell breakdown. Because YO-PRO-1 enters only cells with compromised plasma membranes in early apoptosis, whereas PI labels only late-apoptotic and necrotic cells, the green (YO-PRO-1-positive/PI-negative) and red (PI-positive) populations in Figure 5 correspond to early- and late-stage events, respectively, and can be distinguished directly from the images.

### 2.5. Transcriptomic and Proteomic Analysis of 5637 Cells After ORI Treatment

Transcriptomic and proteomic analyses were performed on 5637 cells treated with ORI (8.142 μM, the 24-h IC50 for 5637 cells). Principal component analysis (PCA) revealed clear separation between control and ORI-treated groups in both the transcriptomic (Figure 6A: PC1 = 46.59%, PC2 = 18%) and proteomic (Figure 6B: PC1 = 59.98%, PC2 = 7.38%) profiles, indicating substantial ORI-induced alterations at the gene- and protein-expression levels. Volcano-plot analysis identified 2783 up-regulated and 2954 down-regulated genes in the transcriptome (Figure 6C) and 1300 up-regulated and 1923 down-regulated proteins in the proteome (Figure 6D), based on statistically defined thresholds (Padj < 0.01). A Venn diagram (Figure 6E) illustrated the overlap between differentially expressed genes (DEGs) and proteins (DEPs), with 1111 molecules significantly altered at both levels. KEGG pathway-enrichment analysis (Figure 6F) revealed significant enrichment of multiple pathways, including “AGE-RAGE signaling pathway in diabetic complications”, “Colorectal cancer”, “Apoptosis”, “Alcoholic liver disease”, “Cell cycle”, and “Bladder cancer”.

### 2.6. ORI Regulates the Expression of Apoptosis-Related Proteins in 5637 and T24 Cells

Following 24 h of exposure to ORI at the 24-h IC50 (8.142 μM for 5637; 9.359 μM for T24), the protein levels of the pro-apoptotic factors BAX (Bcl-2-associated X protein), CASP3 (caspase-3), BID (BH3-interacting-domain death agonist), and CYCS (cytochrome c) were significantly elevated in both 5637 and T24 cells compared with control cells, as confirmed by densitometric quantification (Figure 7A–D). Conversely, the expression of the anti-apoptotic protein BCL2 (B-cell lymphoma 2) was significantly reduced in both cell lines after ORI treatment. β-Actin was used as a loading control.

### 2.7. Anti-Tumorigenic Effects of ORI on 5637 Cells In Vivo

The anti-tumor efficacy of ORI was further investigated in a 5637 xenograft model in nude mice (Figure 8). ORI (20 mg/kg in 200 μL of 5% DMSO in PBS) was administered daily by gavage; control mice received an equal volume of vehicle (5% DMSO in PBS). Analysis of tumor volumes (Figure 8B) revealed a statistically significant reduction in the ORI-treated group compared with the control group (*p* < 0.001). This result was consistent with macroscopic observation, which showed visibly smaller tumors in ORI-treated mice (Figure 8A).

## 3. Discussion

This study provides strong evidence for the anti-bladder-cancer efficacy of ORI, demonstrated through both in vitro and in vivo models. Our main results show that ORI inhibited the proliferation, migration, and invasion of 5637 and T24 bladder cancer cells. In addition, ORI induced S-phase cell-cycle arrest and promoted apoptosis. These effects are closely related to the ORI-mediated regulation of apoptosis-related proteins, manifested by up-regulation of BAX, CASP3, BID, and CYCS and down-regulation of BCL2. Transcriptomic and proteomic analyses confirmed these findings, revealing significant changes in gene and protein expression in pathways critical for cell-cycle regulation and apoptosis. Integrated analysis of the two omics datasets identified 1111 molecules significantly altered at both the mRNA and protein levels (Figure 6E), with concordant enrichment of apoptosis- and cell-cycle-related pathways (Figure 6F). In vivo, ORI significantly inhibited the growth of 5637 xenografts.

Our findings align with previous reports on the anticancer properties of ORI in other malignancies, such as human laryngeal, gastric, and lung cancers, and extend this evidence to bladder cancer. Across numerous studies, ORI has been shown to inhibit cell proliferation and induce apoptosis through various mechanisms, including endoplasmic-reticulum stress, reduced inflammation, and modulation of key signaling pathways. Specifically, Kou et al. [21] reported that ORI induces apoptosis of laryngeal carcinoma cells via endoplasmic-reticulum stress. For instance, Chen et al. [23] found that ORI suppresses the proliferation, migration, and invasion of lung cancer cells by down-regulating AFAP1-AS1. Gao et al. [22] reported that ORI inhibits the proliferation of gastric cancer cells and induces nuclear fragmentation by modulating the TNF-α/AR/TGF-β signaling axis. Liu et al. [25] showed that ORI suppresses the malignant progression of thyroid cancer by inactivating the JAK2/STAT3 pathway, identifying JAK2 as a potential therapeutic target. Jiang et al. [26] found that ORI induces mitochondria-dependent apoptosis in esophageal cancer cells by inhibiting the PI3K/AKT/mTOR and Ras/Raf signaling pathways. Consistent with these findings, our results show up-regulated expression of pro-apoptotic proteins (BAX, CASP3, BID, CYCS) and down-regulated expression of the anti-apoptotic protein BCL2, further confirming that the regulation of apoptosis is one of the core mechanisms by which ORI exerts its anti-tumor effects on bladder cancer. Notably, Liu et al. [24] recently reported that ORI inhibits bladder cancer survival and immune escape by covalently targeting hexokinase 1 (HK1), a mechanism complementary to the apoptosis- and cell-cycle-centred effects identified in our study; together, these findings begin to delineate the multi-faceted mode of action of ORI in bladder cancer. Overall, our study complements existing knowledge by providing evidence of the efficacy of ORI in bladder cancer and proposes potential mechanisms of action applicable to various cancer types.

The strength of this study lies in its comprehensive evaluation of the anti-tumor effects of ORI, integrating in vitro experiments, in vivo experiments, and transcriptomic and proteomic analyses. However, several limitations should be considered. The study relies primarily on two bladder cancer cell lines (T24 and 5637), which may not fully represent the heterogeneity observed in clinical bladder cancer. In addition, although ORI induced a robust and statistically significant S-phase arrest in both cell lines (Figure 2), the key molecular regulators driving this phenotype (e.g., cyclin A2, CDK2, p21 and p27) were not experimentally validated and remain to be identified; Western blot validation of these regulators has been included as a priority experiment in our ongoing follow-up study. Likewise, mitochondrial membrane potential (ΔΨm) depolarization, an upstream event linking BAX/BCL2 imbalance to cytochrome c release, was not directly measured in this work; direct ΔΨm detection (e.g., JC-1 or TMRM staining) will be incorporated into our follow-up mechanistic studies. The precise molecular mechanisms underlying the action of ORI require further in-depth investigation, and its efficacy and safety in humans must be validated through clinical trials.

## 4. Materials and Methods

### 4.1. Reagents and Cell Culture

Oridonin (ORI; B20310) was purchased from Shanghai Yuanye Bio-Technology Co., Ltd. (Shanghai, China). T24 cells ((STCC11701P Servicebio Technology Co., Ltd. Wuhan, China)) were cultured in McCoy’s 5A medium containing 10% FBS and 1% penicillin–streptomycin (PS). 5637 cells (STCC11703P Servicebio Technology Co., Ltd. Wuhan, China) were cultured in RPMI-1640 medium containing 10% FBS and 1% PS. Cells were maintained at 37 °C in a humidified atmosphere with 5% CO2. McCoy’s 5A medium, RPMI-1640 medium, antibiotics (penicillin and streptomycin), phosphate-buffered saline (PBS), trypsin-EDTA, and fetal bovine serum (FBS) were purchased from Wuhan Servicebio (Servicebio Technology Co., Ltd. Wuhan, China). The following reagents were also used: Cell Counting Kit-8 (CCK-8; CA1210), bicinchoninic acid (BCA) protein assay kit (PC0020), and phenylmethylsulfonyl fluoride (PMSF; FJP0100), all from Solarbio (Solarbio Science & Technology Co., Ltd. Beijing, China); an apoptosis and necrosis detection kit (C1075S; Beyotime Biotechnology, Shanghai, China); radioimmunoprecipitation assay (RIPA) lysis buffer (PC104-2) and an enhanced-chemiluminescence (ECL) kit (SQ101), both from Epizyme (Epizyme Biomedical Technology Co., Ltd. Shanghai, China).

### 4.2. Cell Proliferation and Cytotoxicity Assay

Cell proliferation was assessed using the CCK-8 assay. Cells were seeded in 96-well plates at a density of 5 × 10^3^ cells/well (100 μL of cell suspension per well) and pre-incubated for 24 h at 37 °C with 5% CO2. 5637 and T24 cells were then treated with 0, 2.5, 5, 10, 12.5, 15, 17.5, or 20 μM ORI for 24 or 48 h. Subsequently, 10 μL of CCK-8 solution was added to each well (avoiding bubbles), and the plates were incubated for 3 h. Absorbance at 450 nm was measured using a microplate reader. Cell viability and IC50 values were determined from the optical-density values. Each concentration was tested in six replicate wells (*n* = 6).

### 4.3. Wound-Healing Assay

Migration capacity was evaluated using a wound-healing assay. T24 and 5637 cells (5 × 10^5^ cells/well) were cultured in 6-well plates to 90% confluence and then treated with serum-free medium for 12 h. A scratch was made at the bottom of each well with a 200 μL pipette tip, and the wells were washed three times with PBS to remove non-adherent cells. After 24 h of ORI treatment at the 24-h IC50 (8.142 μM for 5637; 9.359 μM for T24), images of the scratched area were obtained using an inverted microscope, and the scratch area was calculated using ImageJ 1.53a software. The experiment was repeated three times.

### 4.4. Transwell Invasion Assay

For the Transwell assay, T24 and 5637 cells (2 × 10^4^ cells/well) were seeded into the upper chamber of Matrigel-coated Transwell inserts. The lower chamber was filled with 600 μL of culture medium containing 10% FBS. After 24 h of ORI treatment at the 24-h IC50 (8.142 μM for 5637; 9.359 μM for T24), cells that had migrated to the lower membrane surface were fixed with 4% paraformaldehyde, stained with crystal violet, and counted under an optical microscope using ImageJ software. The experiment was repeated three times (*n* = 3).

### 4.5. Cell-Cycle Analysis

T24 and 5637 cells were seeded into 6 cm dishes and treated with ORI at their respective IC50 concentrations (9.359 μM for T24 cells and 8.142 μM for 5637 cells). Control cells received an equivalent volume of dimethyl sulfoxide (DMSO). After 24 h of incubation at 37 °C with 5% CO2, the cells were harvested and fixed in 70% ice-cold ethanol at −20 °C overnight. The fixed cells were centrifuged at 1000× *g* for 10 min, washed with PBS, and resuspended. Cells were then stained in the dark with RNase A (1 mg/mL) and propidium iodide (PI; 100 μg/mL) at 37 °C for 30 min. Cell-cycle distribution was analyzed using an Accuri C6 flow cytometer (BD Biosciences, Franklin Lakes, NJ, USA), and the proportions of cells in the G1, S, and G2 phases were determined using FlowJo 7.6.1 software (FlowJo LLC, Ashland, OR, USA). The experiment was repeated three times (*n* = 3).

### 4.6. Apoptosis and Necrosis Assay

Apoptosis and necrosis were detected using the YO-PRO-1/PI apoptosis and necrosis detection kit. 5637 and T24 cells were seeded in 6-well plates and divided into a blank control group and an ORI-treated group (ORI at the 24-h IC50: 8.142 μM for 5637; 9.359 μM for T24); both were incubated at 37 °C with 5% CO2 in a humidified atmosphere for 24 h. The medium was aspirated, and cells were washed once with PBS. An appropriate volume of YO-PRO-1/PI working solution (1 mL per well of a 6-well plate) was added, and the cells were incubated in the dark at 37 °C for 20 min. Fluorescence staining was then observed under a fluorescence microscope. The experiment was repeated three times (*n* = 3).

### 4.7. Western Blot Analysis

5637 and T24 cells were treated with ORI at the 24-h IC50 (8.142 μM for 5637; 9.359 μM for T24) for 24 h. Total protein was extracted on ice using RIPA lysis buffer supplemented with protease inhibitors, and protein concentration was determined with a BCA protein assay kit. Equal amounts of protein were separated by SDS-PAGE and electrophoretically transferred onto polyvinylidene fluoride (PVDF) membranes (Millipore, Billerica, MA, USA). The membranes were blocked with blocking buffer (PS108; Epizyme, Shanghai, China) for 15 min at room temperature and then incubated overnight at 4 °C with the following primary antibodies from Proteintech (Wuhan, China): BAX (50599-2-Ig), CYCS (10993-1-AP), BID (10988-1-AP), CASP3 (19677-1-AP), and β-actin (81115-1-RR); an antibody against BCL2 (bs-0032R; Bioss, Beijing, China) was also used. After washing three times with TBST, the membranes were incubated with an HRP-conjugated goat anti-rabbit IgG secondary antibody (bs-0295G-HRP; Bioss) for 1 h at room temperature. After three additional washes with TBST, protein bands were visualized using an ECL reagent kit (SQ101; Epizyme) and quantified using ImageJ software, with β-actin as the loading control. The experiment was repeated three times (*n* = 3).

### 4.8. RNA Sequencing and Analysis

Total RNA was extracted from 5637 cells treated with ORI (8.142 μM, the 24-h IC50 for 5637 cells) or vehicle control for 24 h at 70–80% confluence. Cells were washed with RNase-free PBS, harvested, and centrifuged at 500× *g* for 5 min at 4 °C. The cell pellets were lysed in 1 mL of TRIzol reagent (≤10^7^ cells) and pipetted repeatedly until clear lysates were obtained. Samples were stored at −80 °C and shipped on dry ice to Metware Biotechnology Co., Ltd. (Wuhan, China) for RNA sequencing. Three independent biological replicates were prepared per group. Differentially expressed genes (DEGs) were identified using DESeq2 with thresholds of an adjusted *p*-value < 0.01 and an absolute log2 fold change >1. Pathway-enrichment analysis was performed on the Metware Cloud platform (https://cloud.metware.cn) with reference to the Gene Ontology (GO) and Kyoto Encyclopedia of Genes and Genomes (KEGG) databases.

### 4.9. Proteomic Sequencing and Analysis

5637 cells were cultured in RPMI-1640 medium supplemented with 10% FBS and 1% penicillin–streptomycin. At 70–80% confluence, cells were treated with ORI at 8.142 μM (the 24-h IC50 for 5637 cells) or with DMSO as a control for 24 h. Cells were washed with RNase-free PBS, harvested, and centrifuged at 500× *g* for 5 min at 4 °C. After discarding the supernatant, samples were stored at −80 °C and shipped on dry ice to Metware Biotechnology Co., Ltd. (Wuhan, China) for proteomic sequencing. Three independent biological replicates were prepared per group. The false-discovery rate (FDR) was controlled at <1% at both the protein and precursor-ion levels. Differentially expressed proteins (DEPs) were identified using thresholds of an adjusted *p*-value < 0.05 and a fold change ≥1.5. Pathway-enrichment analysis was performed on the Metware Cloud platform (https://cloud.metware.cn) with reference to the GO and KEGG databases.

### 4.10. Xenograft Anti-Tumor Assay

All animal experiments were conducted in strict accordance with the ethical guidelines of Tarim University and were approved by the Science and Technology Ethics Committee of Tarim University (protocol code 2024071). Male BALB/c nude mice (6 weeks old, 19–21 g) were purchased from Sipeifu Biotechnology Co., Ltd. (Beijing, China) and used to establish subcutaneous xenograft models. Each mouse received a subcutaneous injection of 5 × 10^6^ 5637 cells in the right axillary region. When tumors reached approximately 50 mm^3^, the mice were randomly assigned to two groups (*n* = 5 per group): an ORI-treatment group (20 mg/kg) and a control group (5% DMSO in PBS). The ORI group received ORI (20 mg/kg in 200 μL of 5% DMSO in PBS) daily by gavage, while the control group received the vehicle (200 μL of 5% DMSO in PBS) daily by gavage. Tumor dimensions were measured with calipers, and tumor volume was calculated as V = (length × width^2^)/2. At the experimental endpoint, the mice were euthanized, and the tumors were excised, weighed, measured, and photographed.

### 4.11. Statistical Analysis

All experiments were performed independently at least three times, and data are presented as the mean ± standard deviation (SD). Data normality was assessed using the Shapiro–Wilk test, and homogeneity of variance using Levene’s test; all data met the assumptions for parametric testing. Statistical analyses were performed using GraphPad Prism 9.5 (GraphPad Software, San Diego, CA, USA), employing one-way analysis of variance (ANOVA) or Student’s t-test, as appropriate. A *p*-value < 0.05 was considered statistically significant.

## 5. Conclusions

In conclusion, this study demonstrates that oridonin (ORI) suppresses bladder cancer cell proliferation, migration, and invasion by inducing S-phase cell-cycle arrest and apoptosis. Integrated transcriptomic and proteomic analyses further revealed that ORI exerts its anti-tumor effects by inducing extensive dysregulation of transcriptional networks and metabolic pathways. These findings provide a mechanistic basis for considering ORI a promising candidate for the treatment of bladder cancer and support the further preclinical development of ORI-based therapeutic strategies.

## Figures and Tables

**Figure 1:**
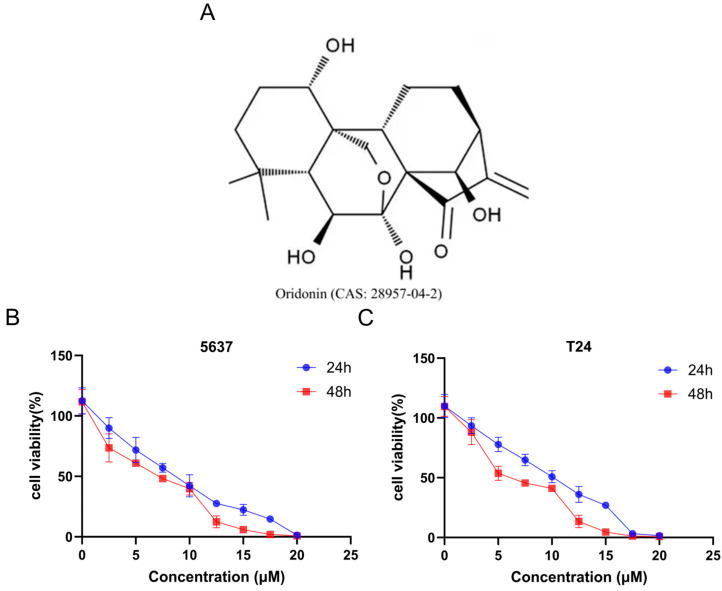
Oridonin (ORI) inhibits the growth of 5637 and T24 cells. Cell viability was assessed using the CCK-8 assay following treatment with ORI (0, 2.5, 5, 10, 12.5, 15, 17.5 and 20 μM) for 24 and 48 h. (**A**) Chemical structure of ORI (CAS: 28957-04-2). (**B**) Dose–response curves illustrating the effect of ORI on 5637 cell viability at 24 h (blue circles) and 48 h (red squares). (**C**) Dose–response curves showing the effect of ORI on T24 cell viability at 24 h (blue circles) and 48 h (red squares). Data are expressed as the mean ± standard deviation (*n* = 6).

**Figure 2:**
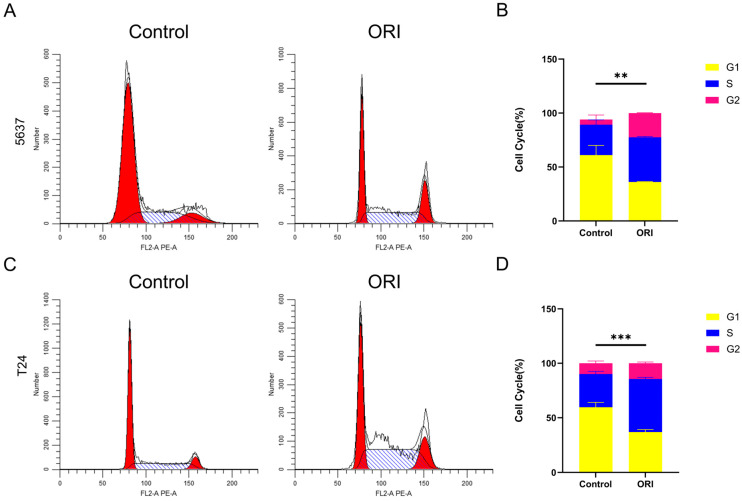
Effect of ORI on cell-cycle progression in 5637 and T24 cells, examined by flow cytometry after 24 h of treatment with ORI at the 24-h IC50 (8.142 μM for 5637; 9.359 μM for T24). (**A**,**C**) Representative histograms of the cell-cycle distribution in 5637 and T24 cultures treated with ORI or vehicle control (DMSO). (**B**,**D**) Bar graphs quantifying the percentage of 5637 and T24 cells in each cell-cycle phase (G1, S, and G2) after exposure to ORI or DMSO. Data represent the mean ± SD from three independent experiments (*n* = 3); statistical significance was determined by Student’s *t*-test. ** *p* < 0.01; *** *p* < 0.001.

**Figure 3:**
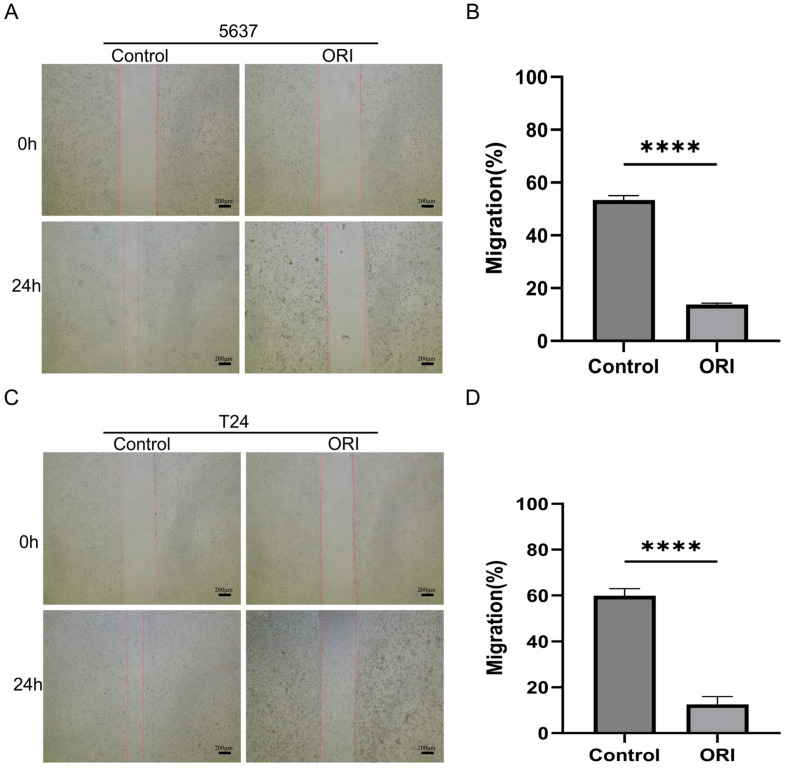
Wound-healing assays showing that ORI inhibits the migratory capacity of 5637 and T24 cells. (**A**) Representative images of wound closure in 5637 cells at 0 and 24 h after scratching, observed by inverted microscopy (40×), treated with control or ORI at the 24-h IC50 (8.142 μM for 5637; 9.359 μM for T24). (**B**) Quantification of 5637 cell migration (%) after control or ORI treatment. (**C**) Representative images of wound closure in T24 cells at 0 and 24 h after scratching (40×). (**D**) Quantification of T24 cell migration (%). Data represent the mean ± SD from three independent experiments (*n* = 3); statistical significance was determined by Student’s t-test. **** *p* < 0.0001. The migration (wound-closure) rate was calculated as (A0 − A24)/A0 × 100%, where A0 and A24 are the scratch areas at 0 and 24 h. Scale bars = 200 μm.

**Figure 4:**
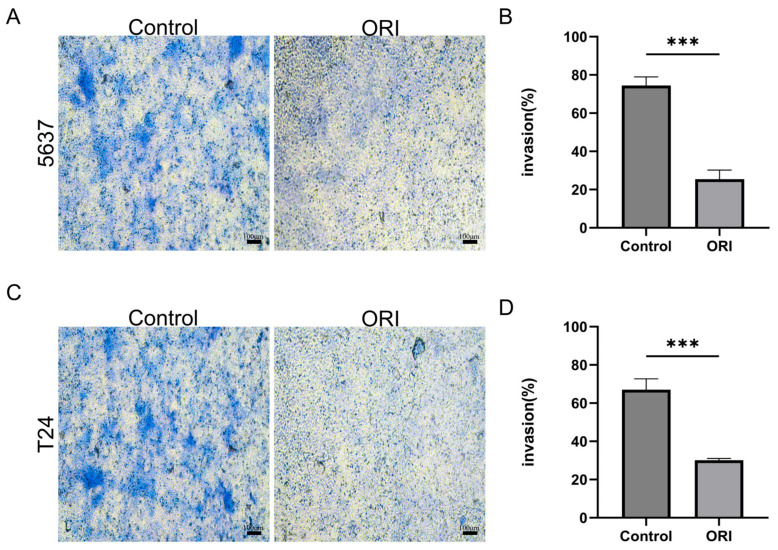
Transwell invasion assays showing that ORI reduces the invasive capacity of 5637 and T24 cells. (**A**) Representative images of invaded 5637 cells under control and ORI-treated (24-h IC50: 8.142 μM for 5637; 9.359 μM for T24, 24 h) conditions. (**B**) Quantification of the percentage of 5637 cells invading through the membrane. (**C**) Representative images of invaded T24 cells under control and ORI-treated (24-h IC50: 8.142 μM for 5637; 9.359 μM for T24, 24 h) conditions. (**D**) Quantification of the percentage of T24 cells invading through the membrane. Cells were fixed with 4% paraformaldehyde for 15 min at room temperature, rinsed three times with PBS (5 min each), stained with 0.1% crystal violet for 5 min, and washed with distilled water. Data represent the mean ± SD from three independent experiments (*n* = 3); statistical significance was determined by Student’s *t*-test. *** *p* < 0.001. The invasion rate was calculated as the percentage of invaded cells relative to the control group. Scale bars = 100 μm.

**Figure 5:**
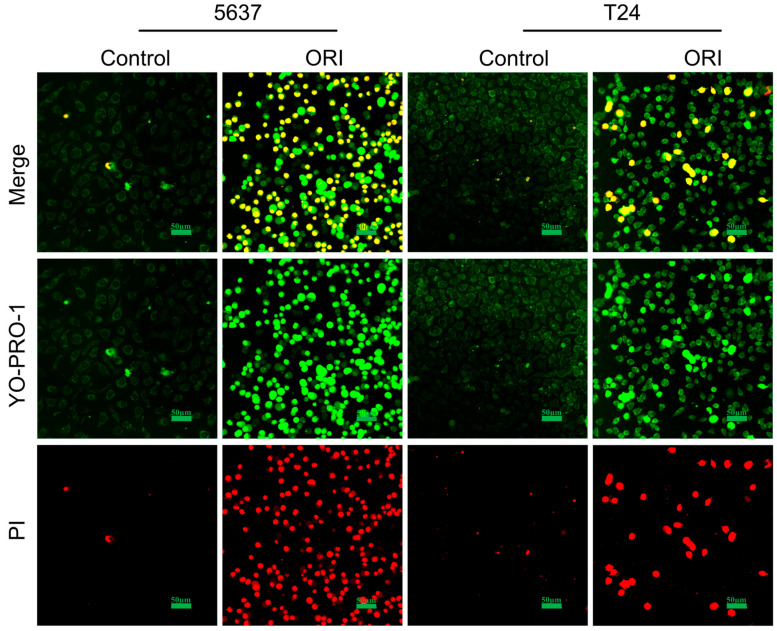
Visualization of apoptosis and necrosis induced by ORI in 5637 and T24 cells. After 24 h of treatment with control or ORI at the 24-h IC50 (8.142 μM for 5637; 9.359 μM for T24), cells were stained with YO-PRO-1 (green, early-apoptotic marker) and PI (red, late-apoptotic/necrotic marker). Fluorescence-microscopy images show representative fields of merged, YO-PRO-1, and PI signals in 5637 and T24 cells. Scale bar = 50 μm (*n* = 3). Early-apoptotic cells are YO-PRO-1-positive/PI-negative (green), whereas late-apoptotic and necrotic cells are PI-positive (red), and yellow signals in the merged images indicate co-localization of YO-PRO-1 and PI.

**Figure 6:**
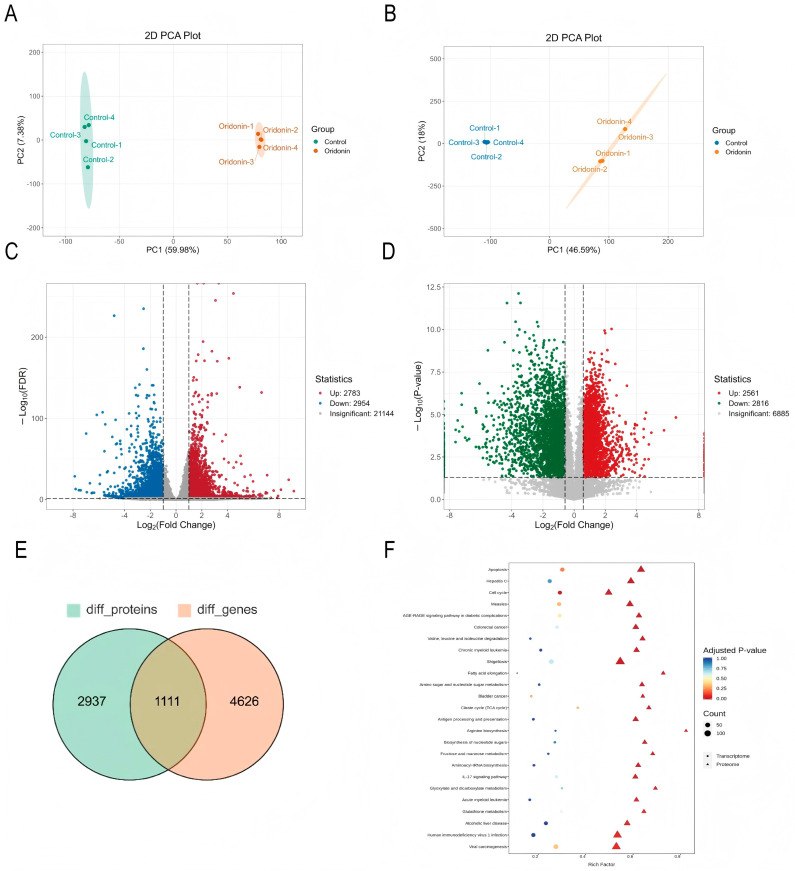
ORI treatment modulates gene and protein expression in 5637 cells. (**A**) Two-dimensional PCA plot of gene (transcriptomic) expression data, showing clear separation between control and ORI-treated samples. (**B**) Two-dimensional PCA plot of protein (proteomic) expression data. (**C**) Volcano plot of gene-expression changes after ORI treatment. (**D**) Volcano plot of protein-expression changes after ORI treatment. (**E**) Venn diagram showing the overlap between differentially expressed genes and proteins. (**F**) Enriched pathways associated with differentially expressed genes and proteins; dot size reflects the number of genes or proteins in the pathway and color indicates the adjusted *p*-value. Circles represent transcriptome data and triangles represent proteome data; the *x*-axis represents the rich factor. Cells were treated with 8.142 μM ORI (the 24-h IC50 for 5637 cells) or vehicle control for 24 h; three biological replicates per group (*n* = 3).

**Figure 7:**
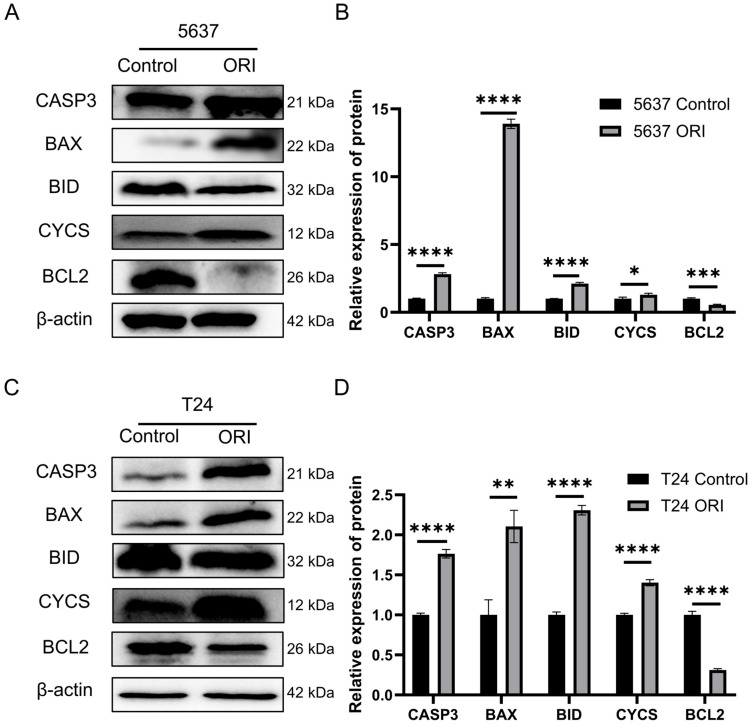
ORI alters apoptosis-related protein profiles in 5637 and T24 cells. The expression of CASP3, BAX, BID, CYCS, and BCL2 was determined by Western blotting following treatment with control or ORI at the 24-h IC50 (8.142 μM for 5637; 9.359 μM for T24) for 24 h; β-actin served as a loading control. (**A**) Representative Western blots for CASP3, BAX, BID, CYCS, BCL2, and β-actin in 5637 cells (control and ORI). (**B**) Densitometric analysis of protein expression relative to β-actin in 5637 cells. (**C**) Representative Western blots in T24 cells (control and ORI). (**D**) Densitometric analysis of protein expression relative to β-actin in T24 cells. Data represent the mean ± SD from three independent experiments (*n* = 3); statistical significance was determined by Student’s *t*-test. * *p* < 0.05, ** *p* < 0.01, *** *p* < 0.001, **** *p* < 0.0001.

**Figure 8:**
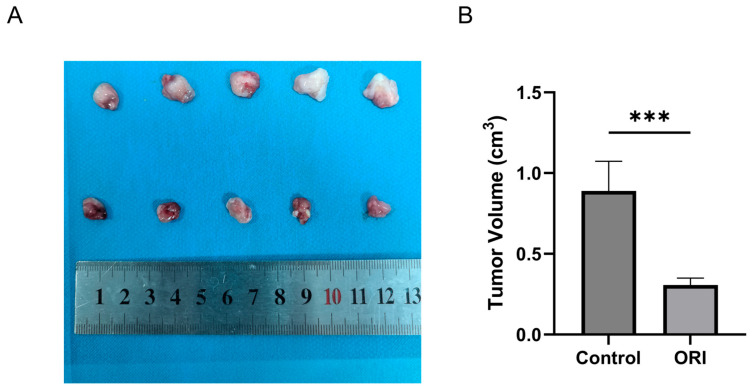
In vivo efficacy of ORI on tumor growth in a nude-mouse model established with 5637 cells. (**A**) Representative images of tumors harvested from mice treated with vehicle control (top) or ORI (bottom). (**B**) Tumor-volume measurements for each group (*n* = 5). Control mice received PBS + 5% DMSO, and the ORI-treated group received ORI (20 mg/kg in 200 μL of 5% DMSO in PBS, daily) by gavage. A significant decrease in tumor volume was observed in the ORI-treated group compared with the control group. Data are shown as the mean ± SD; statistical significance was determined by Student’s *t*-test. *** *p* < 0.001.

## Data Availability

The data presented in this study are available from the corresponding author upon reasonable request.

## Abbreviations

The following abbreviations are used in this manuscript:

ANOVA	Analysis of variance
BC	Bladder cancer
BCA	Bicinchoninic acid
CCK-8	Cell Counting Kit-8
DEG	Differentially expressed gene
DEP	Differentially expressed protein
DMSO	Dimethyl sulfoxide
ECL	Enhanced chemiluminescence
FBS	Fetal bovine serum
FDR	False discovery rate
GO	Gene Ontology
IARC	International Agency for Research on Cancer
IC50	Half-maximal inhibitory concentration
KEGG	Kyoto Encyclopedia of Genes and Genomes
ORI	Oridonin
PBS	Phosphate-buffered saline
PCA	Principal component analysis
PI	Propidium iodide
PS	Penicillin–streptomycin
PVDF	Polyvinylidene fluoride
RIPA	Radioimmunoprecipitation assay
SD	Standard deviation
